# Influence of Accelerating Admixtures on the Reactivity of Synthetic Aluminosilicate Glasses

**DOI:** 10.3390/ma15030818

**Published:** 2022-01-21

**Authors:** Laura Gonzalez-Panicello, Ines Garcia-Lodeiro, Francisca Puertas, Marta Palacios

**Affiliations:** Eduardo Torroja Institute for Construction Science (IETcc-CSIC), 28033 Madrid, Spain; laura.gonzalez@ietcc.csic.es (L.G.-P.); iglodeiro@ietcc.csic.es (I.G.-L.); puertasf@ietcc.csic.es (F.P.)

**Keywords:** synthetic glasses, reaction kinetics, dissolution, accelerating admixtures, reaction products

## Abstract

This research aims at gaining a further understanding of the impact of accelerating admixtures on the reactivity of supplementary cementitious materials (SCMs), which are widely used as a clinker replacement in blended cements. This was done on synthetic glasses with controlled composition and structure that mimic two types of real SCMs (slag and calcium-rich fly ash). The effects of DEIPA, TIPA, NaSCN and Na_2_S_2_O_3_ on the glass dissolution, hydration kinetics and reaction products were investigated. The obtained results concluded that the pH of the NaOH solution and the composition of the synthetic glass play a key role on the effect of the admixtures. In 0.1 M NaOH (pH = 13.0), all the studied admixtures inhibited the dissolution of slag-like glasses while they enhanced the dissolution of Ca-rich fly ash-like glasses, being Na_2_S_2_O_3_ the admixture that led to the highest increase of the dissolution rate of the Ca-rich fly ash-type glasses. In 1 M NaOH solutions (pH = 13.8), only the alkali admixtures (NaSCN and Na_2_S_2_O_3_) enhanced the degree of reaction of both glasses. In slag-type glasses pastes mixed with 1 M NaOH, the addition of 2% Na_2_S_2_O_3_ induced the highest increase of their reactivity as inferred by the total heat release and the amount of bound water. This is related to the formation of a high amount of S(II)-AFm, in addition to C-A-S-H, that would increase the aluminium undersaturation of the pore solution and consequently the further dissolution of the glass.

## 1. Introduction

Clinker replacement by supplementary cementitious materials (SCMs) is currently the most efficient and feasible strategy to decrease the carbon footprint of Portland cement and concrete. Blended cements generally have high long-term strength and durability, however, the substitution of clinker by SCMs has a detrimental impact on the early strength of concrete due to the slow reactivity of the SCMs, which finally limits the level of clinker replacement. Current global levels of clinker substitution are around 30%. Chemical admixtures such as superplasticizers and accelerators have been identified to play a key role to reach higher clinker replacements while preserving the overall mechanical performance [1,2].

Accelerating and strength enhancing admixtures have been widely used to increase the early reactivity and strength of cement and concrete. Their working mechanisms have been extensively investigated in pure Portland cements but barely studied on blended cements. CaCl_2_ is the most effective accelerator of C_3_S and Portland cement. In C_3_S pastes, Juenger et al. [3] concluded that CaCl_2_ enabled the formation of a more permeable C-S-H layer around the cement grains that led to a faster diffusion of water and ions and a higher rate of hydration during the early diffusion-controlled period [3]. Despite its great accelerating effect, the dosage of CaCl_2_ is limited to minimize the risk of steel corrosion in reinforced concrete. Alkalis such as Na_2_SO_4_ and NaOH accelerate the reaction kinetics of alite and cement during the early stages [4,5], however, at equal degree of hydration, the addition of NaOH has shown to decrease the mechanical properties at later ages with respect to plain systems. Mota et al. [4] concluded that NaOH, in contrast to Na_2_SO_4_ inhibited the precipitation of the total volume hydrates, mainly ettringite, with the consequent increase of the porosity and decrease of the strength at these later ages. Alkanolamines such as triisopropanolamine (TIPA), triethanolamine (TEA) or diethanol-isopropanolamine (DEIPA) are effective grinding aids and at certain dosages increase reactivity of cement and the mechanical properties of cementitious materials. TEA has been shown to enhance the reactivity of C_3_A due to the formation of Al^3+^ complex, while it retards C_3_S hydration [6,7]. Furthermore, Gartner and Myers [8] suggested that the complexation of TIPA with Fe^3+^ facilitated iron transport and the enhancement of C_4_AF reactivity.

The impact of accelerating admixtures on SCMs has been generally investigated on blended cements [9,10,11,12]. CaCl_2_ has been shown to increase the reactivity of blast furnace slag after 10 h of hydration in slag-blended cements [13]. In these systems, the formation of AFm had a positive effect on the reduction of the porosity and increase of the mechanical properties but also it would potentially decrease the risk of corrosion because of the chloride binding capacity of the AFm. Alkalis such as sodium oxalate and (K, Na)_2_SiO_3_ increased the reactivity of fly ash in blends (70% fly ash and 30% Portland cement) due to the increase of the pH induced [14]. Alkanolamines have also been reported to enhance the reactivity of aluminosilicate-SCMs. The greater dissolution rate of fly ash in KOH solution (pH = 13) in presence of TEA was explained by its aluminium complexation capacity [15], while studies carried out by Riding et al. [16] suggested that DEIPA increased the reactivity of blast furnace slag in blended cements after 48 h by increasing the attack on the glassy phases or enhancing the mobility of elements dissolving from the slag. Huang et al. [17] concluded from mass balance calculations that TEA and TIPA increased the reactivity of metakaolin in limestone-calcined clay blended cements. While TIPA promoted the early reaction of the metakaolin (at 3 days), TEA had a higher impact at 28 days of reaction. Huang et al. [17] highlighted the relevance of the sulfate content on the impact of the alkanolamines on the early reactivity of metakaolin. In particular, an increase of the sulfates led to the precipitation of ettringite and accelerated dissolution of Al-bearing phases including metakaolin.

This paper aims at gaining further insight into the effect and working mechanism of a series of accelerating admixtures (DEIPA, TIPA, NaSCN and Na_2_S_2_O_3_) on aluminosilicate-SCMs. For this, we have synthesized in the laboratory model glasses that mimic two classes of real SCMs (slag and calcium rich- fly ash) to reduce the complexity of the studied systems and follow the effects on well characterized systems. The influence of different admixtures on their dissolution, hydration kinetics and reaction products were investigated. Hence, the dissolution of both synthetic glasses at far from equilibrium conditions in 0.1 M and 1 M NaOH solution was measured in presence and absence of the admixtures. The reaction kinetics of concentrated suspensions were measured and the reaction products were characterized by techniques such as FTIR, TGA and ^27^Al MAS NMR. This study enabled us to identify the most effective accelerators on model synthetic glasses that can be later exploited to enhance the reactivity of aluminosilicate-SCMs in blended cements and further decrease the clinker content of cement.

## 2. Materials and Methods

### 2.1. Synthesis and Characterization of Synthetic Aluminosilicate Glasses

Two aluminosilicate glasses with chemical compositions similar to blast furnace slag (G1) and calcium-rich fly ash (G2) were synthesized. Their chemical compositions are shown in Table 1. The corresponding stoichiometric quantities of CaCO_3_ (Calcium carbonate, reagent grade, Scharlau, Barcelona, Spain), SiO_2_ (Silica gel, Merck KGaA, Madrid, Spain) and Al_2_O_3_ (Aluminium oxide anhydrous, Merck KGaA) were initially homogenized in ethanol by magnetic stirring (50 g of powder + 250 mL of ethanol). In the case of G1, 5 g pellets were prepared after evaporation of the ethanol and fired at 1500 °C for 4 h in air. Subsequently, the molten sample was quickly quenched in ultrapure water. In the case of G2 glass, after the evaporation of the ethanol, 5 g pellets were pressed and treated at 1600 °C for 4 h. The molten sample was afterwards quickly quenched on a brass plate. This quenching method applied in G2 glasses enabled a better preservation of the platinum crucibles during the glass extraction in comparison with quenching method applied for G1 glasses. As shown by X-ray powder diffraction (XRD) (Advance AXL D8 diffractometer, Bruker, Billerica, MA, USA) in Figure S1 in the Supplementary Material, the quenching method did not affect the structure of the obtained glasses.

**Figure 1:**
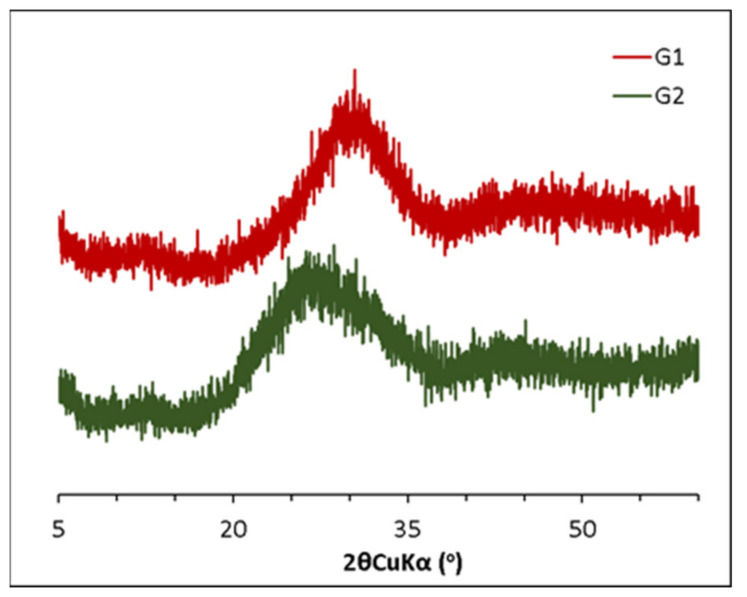
X-ray powder diffraction patterns of the synthetic glasses.

Both glasses were ground in a planetary ball mill. Figure 1 shows the XRD pattern of the synthetic glasses. The absence of Bragg peaks in the XRD patterns confirms the absence of crystalline phases and the lack of long-range order in both synthetic glasses. It is observed that the positions of the amorphous humps are shifted toward greater angles (2θ) with increasing CaO content and the degree of depolymerization. This agrees with the lower polymerization degree of G1 with respect to G2, calculated according to Equation (1) proposed by Mills [18]. It is known that the polymerization degree is associated with the amount of non-bridging oxygen present (NBO) in a glass, given as the mean number of NBO per cation in tetrahedral coordination (NBO/T) and calculated from glass composition in oxide mol fraction (Table 1).

(1) $$\frac{\mathrm{NBO}}{T}=\frac{2\left[\mathrm{CaO}\right]-\left[{\mathrm{Al}}_{2}O_{3}\right]}{\left[{\mathrm{SiO}}_{2}\right]+\left[{\mathrm{Al}}_{2}O_{3}\right]}$$

### 2.2. Dissolution Experiments of the Aluminosilicate Glasses

Dissolution experiments of aluminosilicate glasses in 0.1 M NaOH (pH = 13.0) and 1 M NaOH (pH = 13.8) solutions were carried out in 1L polypropylene containers that were kept in a water-bath controlled at 25 °C. It is worth highlighting that the pH values in presence of concentrations of Na of 1 M to 3 M can underestimate the real pH value by a factor of 0.3 and 0.5 because of the alkali error [19].

The dissolution tests were done following the protocols of previous studies [20,21]. The glass particles were sieved between 45 and 125 µm and then placed inside a nylon meshed basket with a pore size of 0.40 µm suspended in the container with a nylon thread, similar to previous work [20,21]. 0.1 M NaOH and 1 M NaOH solutions (Sodium hydroxide pellets, Scharlau) were prepared using ultra-pure water (18.2 MΩ·cm by a Milli-Q A+ water purification system from Millipore, Merck & Cie, Guyancourt, France). The liquid/solid ratio was 1000 and kept in agitation with a magnetic stirrer over 2 and 7 days for dissolution experiments in 0.1 M NaOH and 1 M NaOH, respectively. Then, 6 mL aliquots of solution were collected at different times. The solution was filtered through a 0.22 µm syringe filter and acidified with 2%wt HNO_3_. The chemical composition of the solutions was analysed by the ICP-OES (VARIAN 725-ES ICP) (Melbourne, Australia). The extracted solution was replaced by another 6 mL of fresh solution NaOH (with or without admixture) to maintain constant the l/s ratio and the admixture dosage. Dissolution tests were carried out in absence and presence of 0.02% DEIPA by weight of dry glass (bwg) and 0.02% bwg TIPA, of 0.3% bwg sodium thiocyanate (NaSCN) and 0.3% and 2% bwg of sodium thiosulfate (Na_2_S_2_O_3_). The accelerating effect of these admixtures at these dosages on the reactivity of blended and non-blended cements have been previously reported in the literature [12,16,22,23,24].

The dissolution rates were calculated considering the Si concentrations during the linear period (first 360 min) by using Equations (2) and (3) [20]:

(2) $$Q_{\mathrm{norm}}=\frac{C_{i}\cdot V}{\mathrm{SSA}\cdot m\;\cdot x_{i}}$$

(3) $$r=\frac{Q_{\mathrm{norm}}}{t}$$

where C_i_ is the concentration of the element in solution (mol/L), V is the volume of NaOH solution (L), SSA is the initial specific surface area of the glass (cm^2^/g), m is the mass of glass (g), and x_i_ is the atomic fraction of the element in the glass [25]. Furthermore, the degree of reaction (DoR) of the glass at the end of the dissolution test was calculated according to Equation (4) using the concentration of Si in solution.

(4) $$\mathrm{DoR}=\frac{C_{i}\cdot V}{m\;\cdot x_{i}}$$

### 2.3. Effect of Accelerators on the Reactivity and Mineralogy of Synthetic Glass Pastes

#### 2.3.1. Preparation of Pastes

G1 and G2 were ground in a planetary ball mill and sieved below 45 µm. Table 2 shows the particle size and the specific surface area (SSA_BET_) of the synthetic glasses. Pastes of the synthetic glasses were prepared by mixing 10 g of the glasses with 4 g of 1 M NaOH solution. Pastes were initially mixed with a vertical mixer (JANKE KUNKEL IKA-WERK RW 20, Staufen, Germany) for 30 s at 200 rpm and afterwards at 840 rpm for 3 min. Then, 0.02% bwg of DEIPA (Sigma Aldrich, Buchs, Switzerland), 0.02% bwg of TIPA (Aldrich Chemistry, St. Louis, MO, USA), 0.3% bwg of NaSCN (Sigma Aldrich, St. Louis, MO, USA) and 2% bwg of Na_2_S_2_O_3_ (Acros Organics, Branchburg, NJ, USA) were added into the mixing liquid.

#### 2.3.2. Hydration Kinetics and Characterization of the Reaction Products

Hydration kinetics were measured by isothermal calorimetry (TAM AIR, TA Instruments, New Castle, DE, USA) at 25 °C, 5 g of paste was loaded in plastic ampoules and placed in the calorimeter for 7 days. For the calculation of the total heat release, the first 30 min were not considered as this is the time that the device needs to reach equilibrium after placing the sample.

The reaction of G1 and G2 pastes was stopped by solvent exchange at different reaction times. Next, 1 g of glass paste was mixed with 10 g of isopropanol for 1 min. The suspension was filtered afterwards through a nylon filter with a pore size of 0.45 μm by Sartorius (Sartorius Stedim Biotech GmbH, Göttingen, Germany). The powder was dried in a desiccator up to constant weight.

The amount of chemically bound water (BW) of the pastes was quantified by thermogravimetric analysis (TGA). TGA experiments were carried out by using a TGA-DCS-DTA Q600 equipment (TA Instruments). Around 40 mg of sample in an alumina (Al_2_O_3_) crucible was heated from 25 °C up to 1000 °C with a rate of 10 °C/min under a 100 mL/min flow of N_2_. The amount of chemically bound water was determined from the weight loss of the sample between 25 °C and 550 °C. Fourier-transform infrared spectroscopy (FTIR) spectra were obtained by using a Nicolet 6700 spectrometer (Thermo Scientific, Madison, USA). KBr pellets were prepared by mixing 1 mg of sample and 200 mg of KBr. Frequencies were scanned in the range of 4000–400 cm^−1^, with a resolution of 4 cm^−1^.

Phase identification in G1, G2 pastes in absence and presence of 2% Na_2_S_2_O_3_ at 7 days of hydration was also done with a D8 ADVANCE diffractometer (BRUKER-AXS, Billerica, MA, USA) in a Θ-2Θ configuration using a Cu-Kα1, α2 (1.5406 Å, 1.5444 Å) radiation. The samples were scanned for 3 h 16 min between 5° and 60°, with a step size 0.01973° and with ultra-fast RX “Lynxeye” detector (includes a slit 3 mm anti-scatter, a 2.5° Soller 2nd slit and a Ni K-beta filter (0.5%)). ^27^Al MAS spectra were recorded with a Bruker AVANCE-400 spectrometer (Karlsruhe, Germany) (9.4T magnetic field). A 4-mm (outer diameter) ZrO_2_ rotor was used at a spinning frequency of 10 kHz. The ^27^Al spectra were obtained at a resonance frequency of 104.3 MHz. π/6 pulses of 2 μs, a recycle delay of 5s and 400 scans were applied during the measurements. Chemical shift values of NMR resonances were referred to 1 M solution of AlCl_3_ aqueous solutions.

## 3. Results

### 3.1. Dissolution of the Aluminosilicate Glasses at Far from Equilibrium Conditions

**Figure 2:**
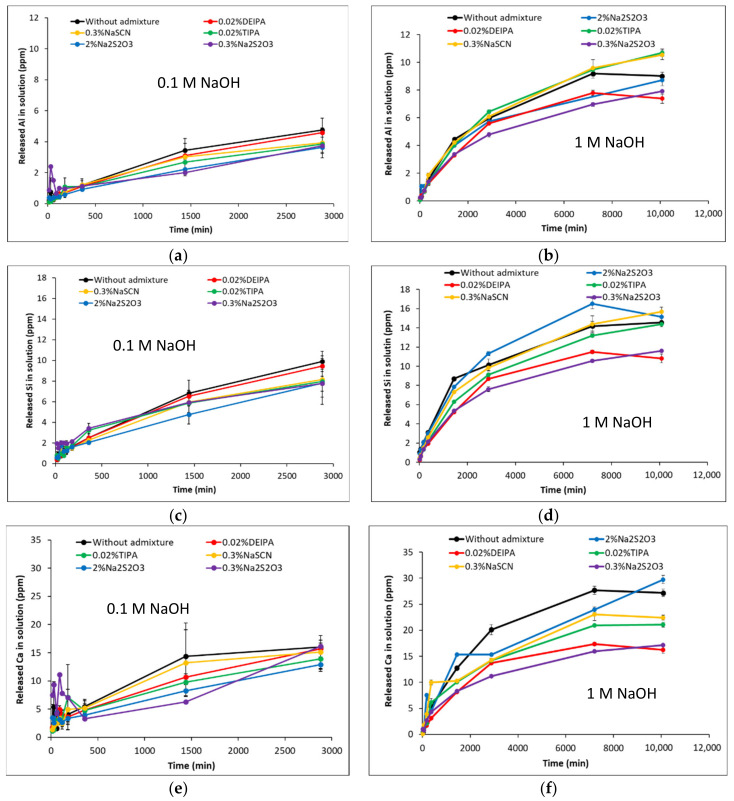
Al, Si and Ca concentrations evolution during the dissolution of G1 glass in (**a**,**c**,**e**) NaOH 0.1 M and (**b**,**d**,**f**) NaOH 1 M in presence and absence of admixture.

**Figure 3:**
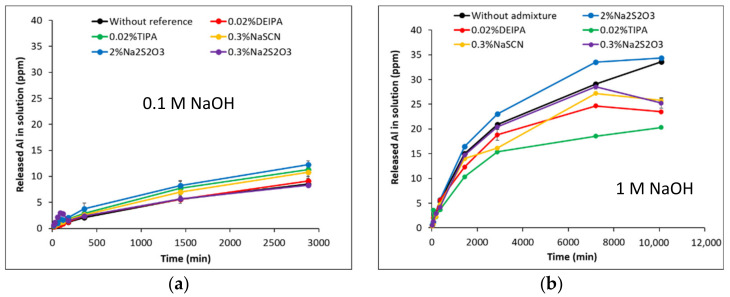

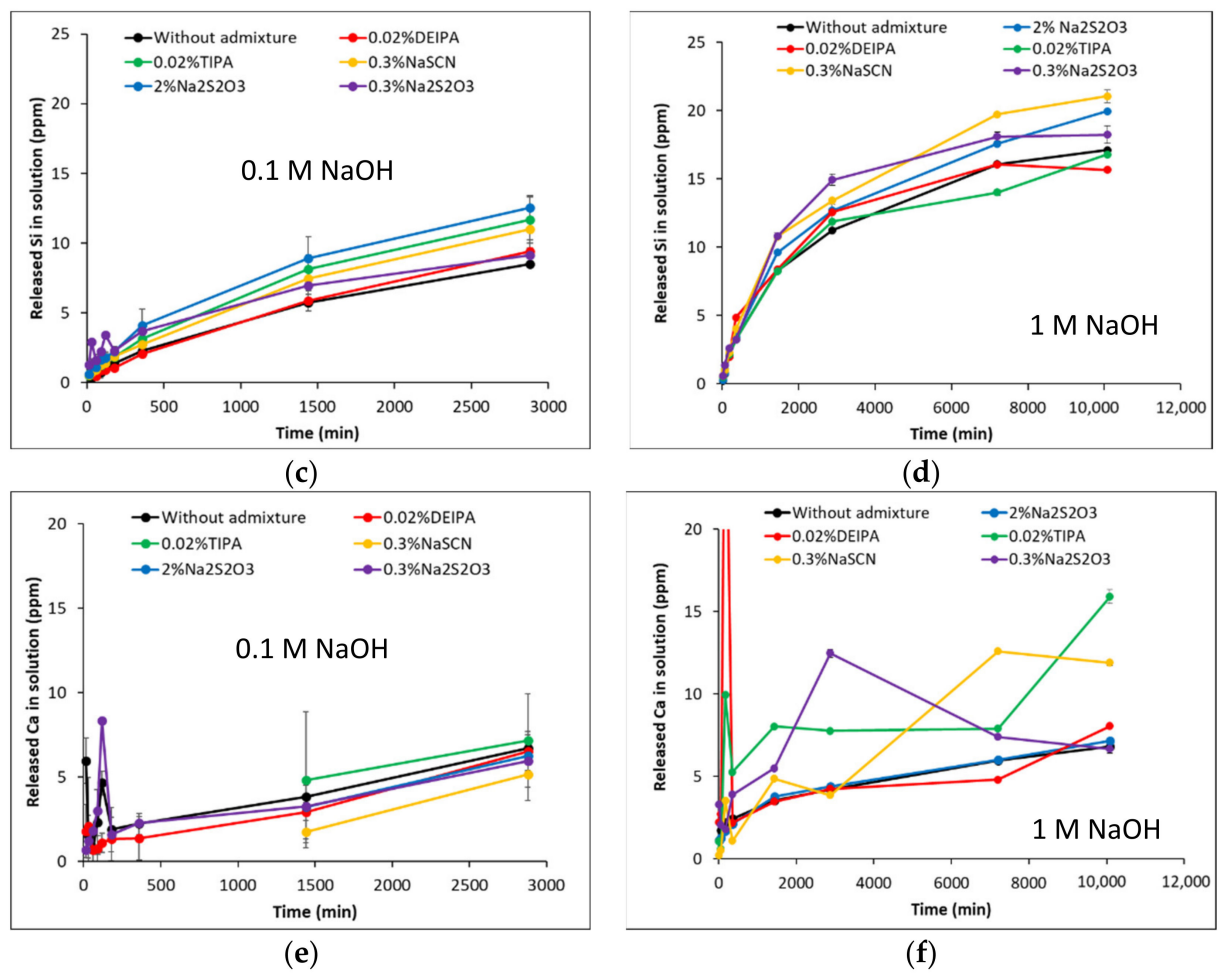
Al, Si and Ca concentrations during the dissolution of G2 glasses in (**a**,**c**,**e**) 0.1 M NaOH and (**b**,**d**,**f**) 1 M NaOH in presence and absence of admixtures. In the dissolution tests of G2 in 0.1 M NaOH containing 0.02% bwg TIPA, 2% bwg Na_2_S_2_O_3_ and 0.3% bwg NaSCN, the initial Ca concentrations were below the ICP detection limit and have not been included.

Figure 2 and Figure 3 show the dissolution of G1 and G2 glasses over time, with and without admixtures, in 0.1 M NaOH and 1 M NaOH solutions. In particular, the concentrations of Al, Si and Ca released into the alkaline solution are presented. For all the studied cases, two different dissolution regimes can be observed for Al and Si. An “initial dissolution” stage over the first 360 min due to the hydrolysis of the network forming species (Al-O-Si and Si-O-Si bonds). As the concentration of the different elements in solution increased, the dissolution rate decreased and a “steady-state dissolution” was reached that can be explained by the local saturation of the solution with respect to the glass or by the formation of alteration layers on the glass surface [26,27]. The amount of Al, Si and Ca released in the dissolution experiments carried out in 0.1 M NaOH were significantly lower (20–60%) with respect to 1 M NaOH, as the reactivity of the glasses increases with the pH of the solution.

**Figure 4:**
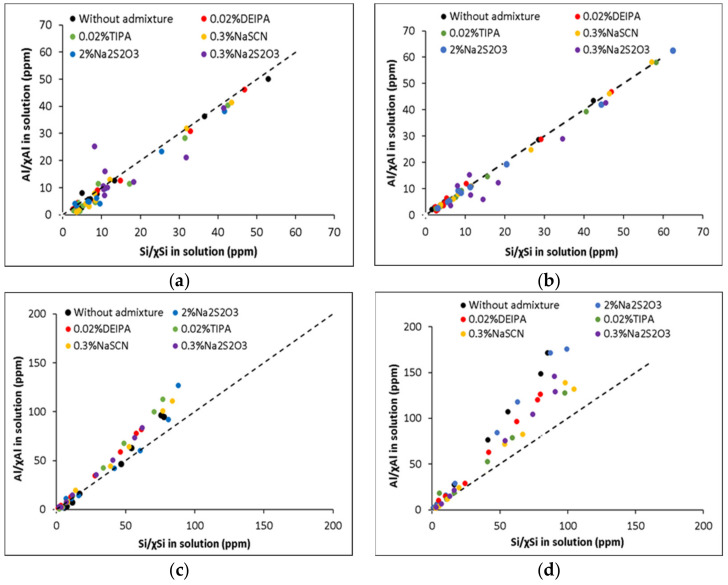
Al concentrations in solution normalized to the molar fraction of Al in the glass plotted in function of the Si normalized concentration of (**a**) G1 in 0.1 M NaOH; (**b**) G2 in 0.1 M NaOH; (**c**) G1 in 1 M NaOH; (**d**) G2 in 1 M NaOH.

Moreover, the pH plays a key role on the mechanism of dissolution of the glasses. In both NaOH solutions, the release of Ca in G1 is preferential over Si and Al previously reported to be due to surface leaching and the depletion in Ca of the glass surface [28] (Figure 4). The lower amount of Ca in G2 with respect to G1 led to lower concentrations of Ca in solution during the dissolution test of the former. In 0.1 M NaOH solution (pH = 13.0), a congruent dissolution of both glasses with respect to Si and Al was observed with no significant preferential release of elements when their concentrations are normalized to their fraction in the glass (Figure 4a,b). However, in 1 M NaOH solution (pH = 13.8), the glasses congruently dissolved (with respect to Al and Si) up to 360 min while a non-congruent dissolution of G-1 and G-2 occurred afterwards. This could be explained by the formation of an alteration layer on the glass surface and possibly to the formation of a reaction product despite the high liquid/solid ratio used during the dissolution experiment (Figure 4c,d).

**Figure 5:**
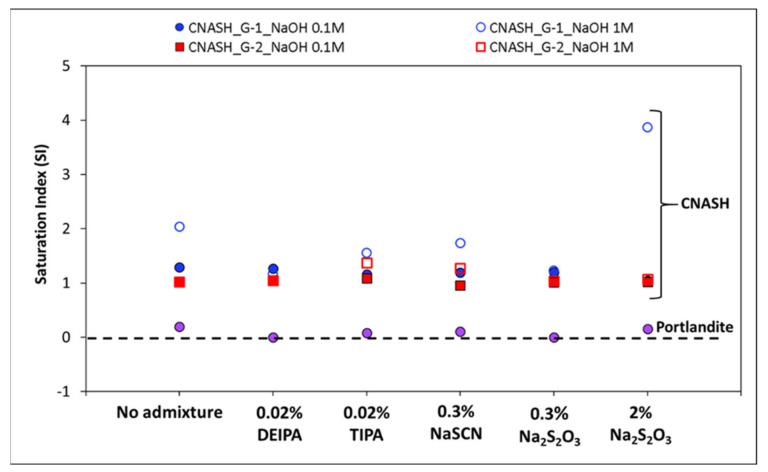
Calculated saturation indices as a function of the type of accelerating admixture, synthetic glass and NaOH concentration (at 2 and 7 days, for 0.1 M NaOH and 1 M NaOH, respectively, when the highest concentration of the elements was measured). A positive saturation index indicates oversaturation.

To explore this possible precipitation of reaction products, the saturation index of different solids was calculated using the measured concentrations of Ca, Al and Si in the dissolution tests at the end of the test, 2 days and 7 days for 0.1 M NaOH and 1 M NaOH, respectively (see Table S1 in the Supplementary Material). A positive saturation index involves oversaturation that indicates that solid could precipitate. For this purpose, the Gibbs Energy Minimization software (GEMS-PSI) [29] and the GEMS-PSI [30] and Cemdata18 [31] thermodynamic databases were used. Saturation index (SI) is defined as log (IAP/KSO), where IAP is the ion activity product calculated from activities derived from the measured concentrations and KSO is the solubility product of the corresponding solid [32]. As shown in Figure 5, positive saturation indices were calculated for C-(N)-A-S-H for G1 and G2 in both NaOH solutions at the end of the dissolution tests, which suggests the possible precipitation of this solid phase. The highest SI is calculated for G1 in 1 M NaOH, mainly in presence of 2% Na_2_S_2_O_3_, which would indicate the higher oversaturation of the solution with respect to C-(N)-A-S-H in presence of this admixture. Furthermore, slightly positive values of SI have been calculated for portlandite for G1 in NaOH, which could indicate its possible precipitation. A further analysis of the glasses by SEM after the dissolution tests confirmed the precipitation of reaction products on the glass surface, mainly in the case of those in contact with 1 M NaOH (see Figure S2 in the Supplementary Material).

For the G1 synthetic glasses, the addition of the accelerating admixtures decreased the amount of Ca, Al and Si released into the solution during the dissolution experiments in 0.1 M NaOH (pH = 13.0) (see Figure 2a,c,e) and decreased up to 1% the degree of reaction of the glass (see Table 3). This would infer that at pH = 13.0, the studied admixtures inhibited the G1 dissolution that could be explained by complex formation or admixture adsorption onto the glass surface [33,34]. In 1 M NaOH (pH = 13.8) the presence of 0.02% DEIPA and 0.3% Na_2_S_2_O_3_, decreased the concentration of the elements in solution over 7 days with respect to the free-admixture sample. The addition of 0.3% NaSCN does not significantly affect the Si and Al release into solution after 5 days, however, at 7 days an increase of their concentrations with respect to the plain sample was observed. The addition of 2% Na_2_S_2_O_3_ enhanced the concentration of Si in solution and the dissolution rate over the first 360 min, while it reduced the amount of Al in solution with respect to the plain sample over the 7 days. Finally, in 1 M NaOH, TIPA decreased the amount of Si released into the solution, it does not modify the amount of Al released during the first 5 days with respect to the free-admixture solutions, but higher Al concentrations were measured at 7 days.

For G2 glasses in 0.1 M NaOH, the addition of 0.3% NaSCN, 2% Na_2_S_2_O_3_ and 0.02% TIPA increased the concentration of Al and Si in solution, and consequently, the degree of reaction of the glasses as shown in Table 3. In particular, the addition of 2% Na_2_S_2_O_3_ increased the degree of hydration after 48 h from 4% (in absence of admixture) up to 6%. The addition of 0.02% DEIPA did not modify the amount of these elements in solution with respect to the plain solution, while 0.3% Na_2_S_2_O_3_ slightly increased the Si concentration in solution but it did not have an effect on the Al concentration. In fact, 0.02% DEIPA and 0.3% Na_2_S_2_O_3_ did not significantly modify the degree of reaction of G2 in 0.1 M NaOH after 48h. In 1 M NaOH (pH = 13.8), NaSCN and Na_2_S_2_O_3_ at the studied concentrations increased the amount of Si released into solution and increased the degree of reaction at 7 days from 8.5% (without admixture) up to 9.9% with both alkali admixtures. However, while 2% Na_2_S_2_O_3_ slightly increased the amount aluminates in solution, 0.3% of Na_2_S_2_O_3_ and 0.3% NaSCN slowed down the Al release. The addition of 0.02% TIPA and DEIPA did not significantly affect the release of Si while decreasing the concentration of Al in solution, and led to a decrease of 0.5–1% of the degree of reaction of G2 after 7 days (Table 3).

### 3.2. Hydration Kinetics of the Glasses

**Figure 6:**
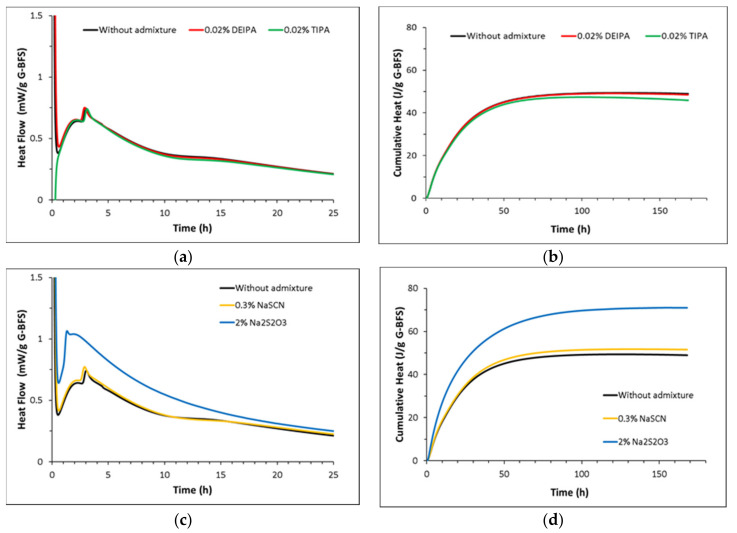
Normalized heat flow and cumulative heat of G1 pastes with (**a**,**b**) without admixture, 0.02% bwg DEIPA, 0.02% bwg TIPA and (**c**,**d**) without admixture, 0.3 bwg %NaSCN, 2% bwg Na_2_S_2_O_3_.

**Figure 7:**
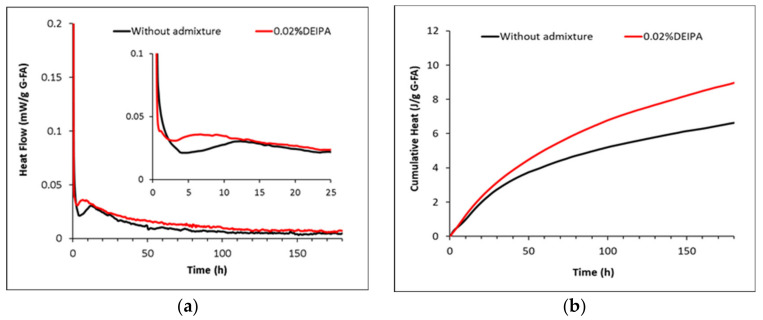

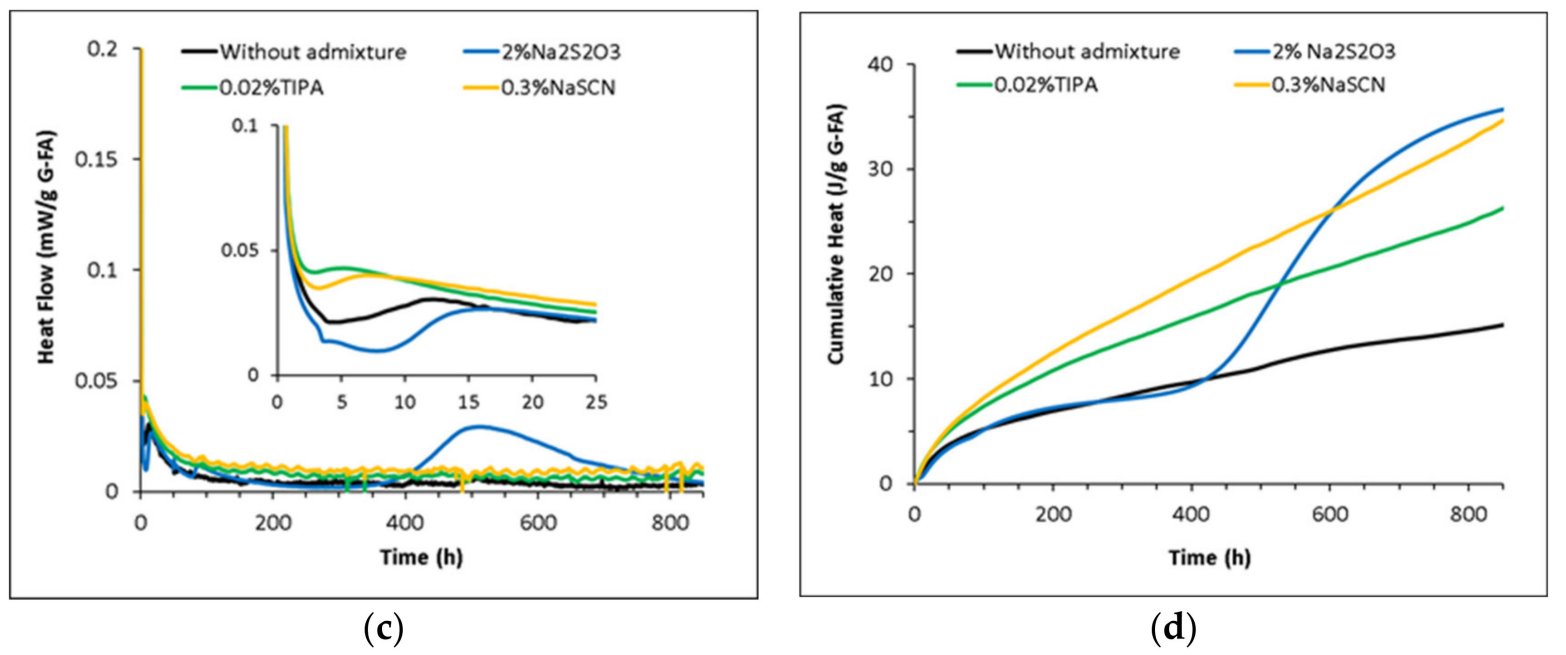
Normalized heat flow and cumulative heat of G2 pastes with (**a**,**b**) without admixture, 0.02% bwg DEIPA, 0.02% bwg TIPA and (**c**,**d**) without admixture, 0.3% bwg NaSCN, 2% bwg Na_2_S_2_O_3_.

Figure 6 and Figure 7 show the heat flow and total heat released during the reaction of the synthetic glasses with 1 M NaOH solution in the presence and absence of the admixtures. G1, with a chemical composition similar to blast furnace slag, shows a higher heat flow than G2, reaching a maximum of 0.75 mW/g at around 3 h. In contrast, a maximum peak of around 0.03 mW/g is observed for G2 glass at around 10 h of reaction. This confirms the lower heat released by glasses with a higher SiO_2_ content, in agreement with Schöler et al. [25].

The addition of 0.02% DEIPA, 0.02% TIPA and 0.3% NaSCN does not have an impact on either, the heat flow or the total heat released in G1 pastes mixed with 1 M NaOH. However, samples containing 2% Na_2_S_2_O_3_ show an increase of the intensity of the main peak from 0.75 to 1 mW/g and of the total heat from 45 J/g to 70 J/g after 7 days of reaction with respect to the plain pastes that confirms the enhancement of the reactivity of the blast furnace slag model glasses with this admixture. This increase of the reactivity of G1 induced by 2% Na_2_S_2_O_3_ in NaOH 1 M is in agreement with the increase of Si in solution measured in the dissolution tests (see Figure 2) and the higher oversaturation with respect to C-(N)-A-S-H calculated by thermodynamic modelling (see Figure 5), although these tests were done in far from equilibrium conditions.

In the case of G2 pastes mixed with 1 M NaOH, the addition of 0.02% DEIPA, 0.02% TIPA and 0.3% NaSCN leads to a progressive increase of the heat released. After 35 days of reaction, the addition of TIPA and NaSCN rises the total heat from 15 J/g (in absence of the admixture) to 26 and 35 J/g, respectively. Pastes containing 2% Na_2_S_2_O_3_ show a similar initial evolution of the total heat release as the admixture-free sample, but this parameter dramatically increases after 16 days of reaction reaching 35 J/g at 35 days. However, the reason behind the retarded increase of the reactivity induced by Na_2_S_2_O_3_ remains unclear.

### 3.3. Mineralogical Characterization of the Glass Pastes

**Figure 8:**
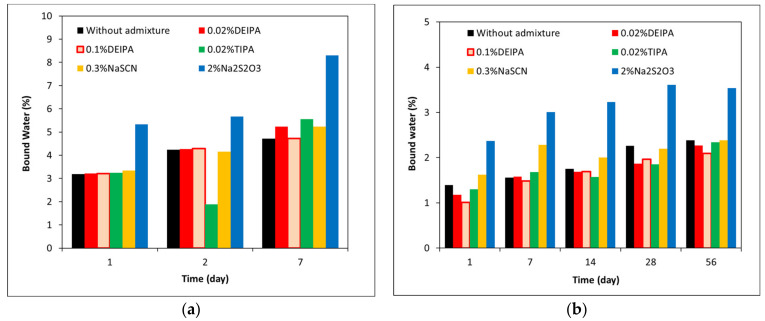
Percentage of chemically bound water in (**a**) G1 and (**b**) G2 mixed with 1 M NaOH with and without accelerating admixtures.

The total chemically bound water content of the pastes of synthetic glass mixed with 1 M NaOH, in the presence and absence of the accelerating admixtures, is shown in Figure 8. G1 pastes showed a higher total bound water than G2 pastes that confirms a higher reactivity of the glass with a lower polymerization degree. For both glasses, Na_2_S_2_O_3_ was the admixture that led to the highest increase of the bound water and consequently the highest amount of reaction products formed.

**Figure 9:**
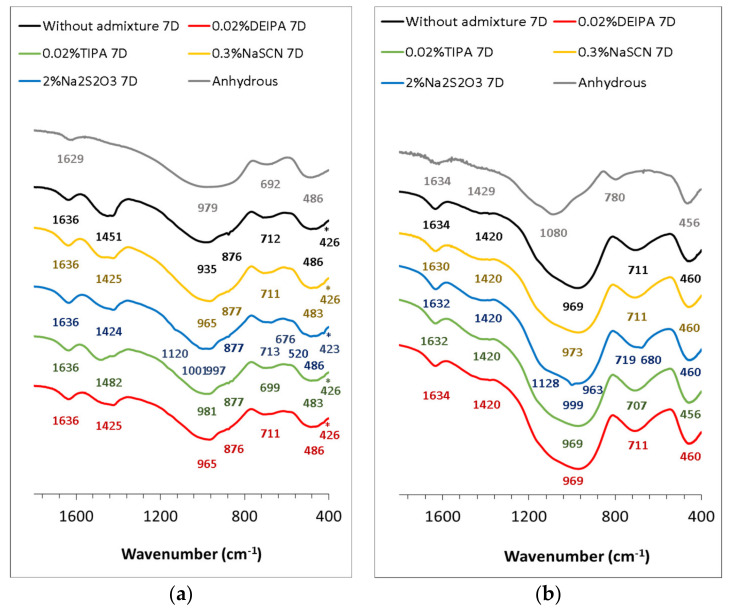
FTIR spectra of 1 M NaOH-synthetic glass pastes at 7 days of reaction, in absence and presence of the accelerating admixtures (0.02% bwg DEIPA, 0.02% bwg TIPA, 0.3% bwg NaSCN, 2% bwg Na_2_S_2_O_3_). (**a**) G1 pastes, (**b**) G2 pastes.

The FTIR spectra for the anhydrous G1 and G2 and the hydrated pastes at 7 days, respectively, are shown in Figure 9. Both anhydrous glasses show a wide vibration band between 1300 and 800 cm^−1^ associated with the Si-O-T (where T is Si or Al) stretching vibrations in the TO_4_ tetrahedra. This band appeared at higher wavenumbers for G2 that confirmed its higher polymerization with respect to G1, as already indicated by XRD and its lower NBO/T (see Figure 1 and Table 1, respectively). The band between 600 and 400 cm^−1^ is attributed to the Si-O-Si and Si-O-Al bending vibrations, while the asymmetric stretching vibration bands assigned to Al-O appeared in the range between 800 and 600 cm^−1^ [35]. The addition of the accelerating admixtures did not significantly modify the FTIR spectra of the hydrated pastes with respect to the plain samples. In all cases, for G1 glasses after 7 days of reaction, the υ_3_ (Si-O) band was narrower and appeared at higher wavenumbers with respect to the anhydrous glasses, which indicates the formation of more polymerized reaction products, in particular, C-(N)-A-S-H. For G2 glasses, υ_3_ (Si-O) shifts from 1080 cm^−1^ (in the anhydrous glass) to 969 cm^−1^ were observed due to the lower polymerization degree of the C-(N)-A-S-H with respect to the starting glass. In G1 pastes containing Na_2_S_2_O_3_, the presence of the vibration bands at 1120, 1001 and 676 cm^−1^ suggested the formation of S(II)- AFm [36].

**Figure 10:**
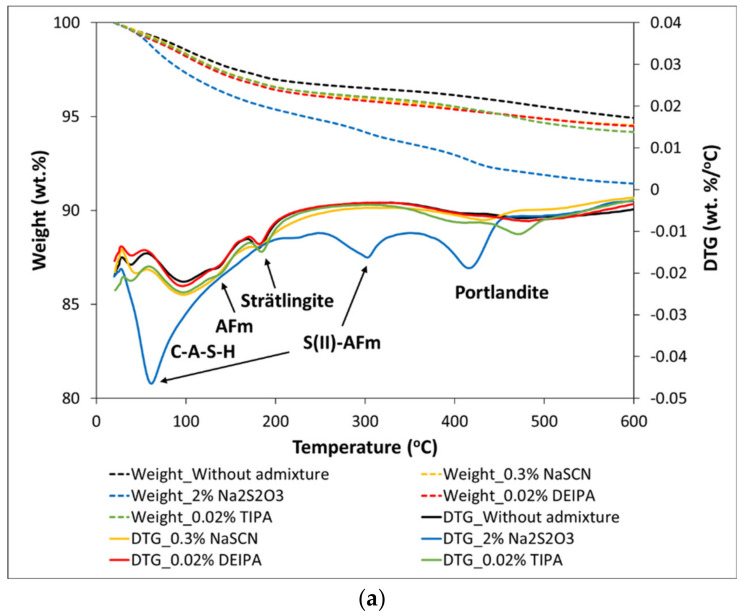

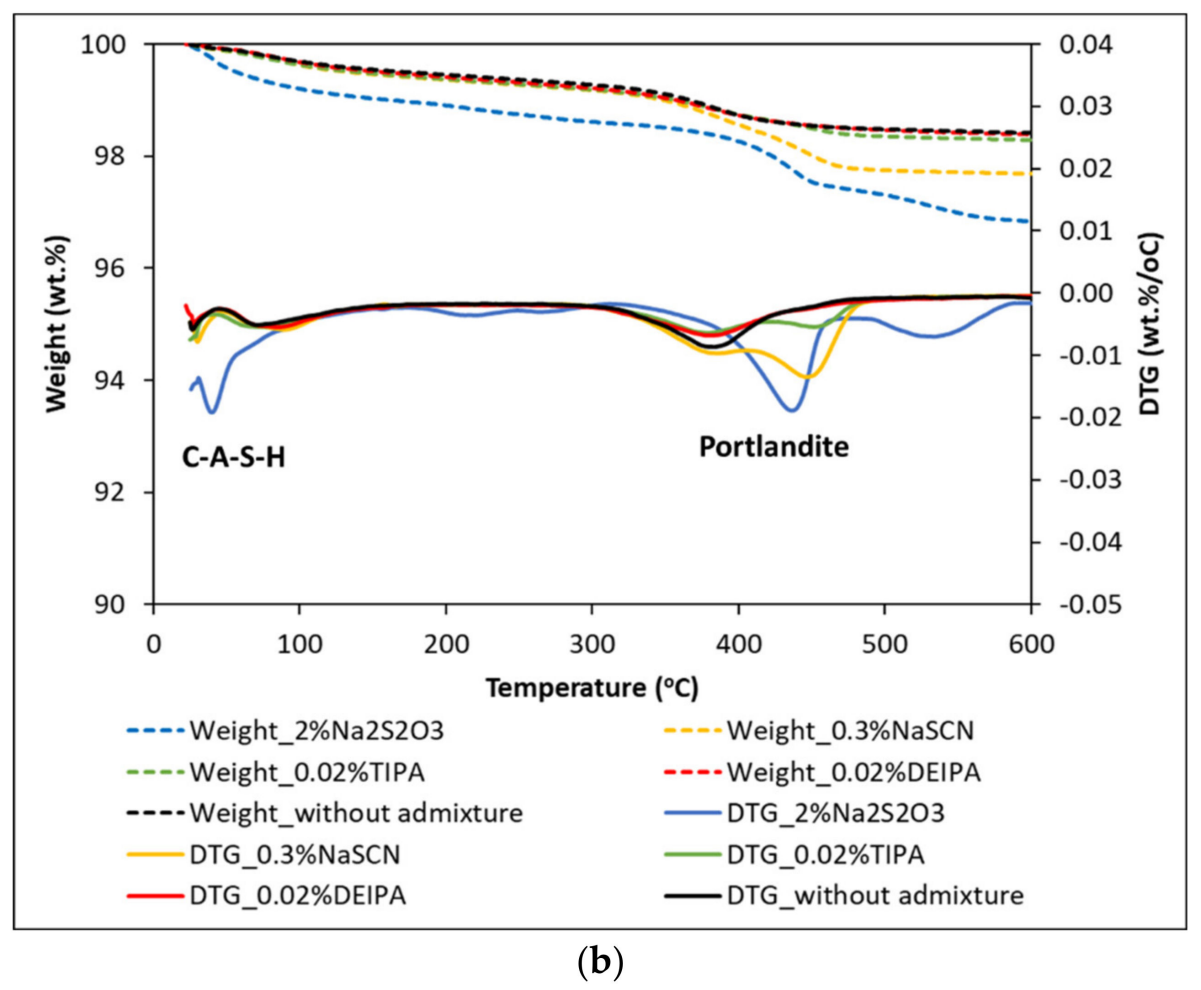
DTG and TGA curves for pastes of (**a**) G1 and (**b**) G2 mixed with 1 M NaOH with and without accelerating admixtures at 7 days of reaction.

For both glasses, the addition of NaSCN, DEIPA and TIPA did not induce significant changes in the type of hydrates formed with respect to plain pastes as observed by TGA in Figure 10. In G1 pastes, the weight loss measured by TGA (Figure 10a) in the range between 50–155 °C was associated with the dehydration of C-A-S-H, and those ocurring between 60–200 °C and 200–300 °C were associated with the decomposition of AFm phases such as calcium monocarboaluminate [37,38], while at 185 °C the decomposition of structural OH of strätlingite was observed [39]. The TGA of G2 pastes, without admixture and with NaSCN, DEIPA and TIPA, only showed the peak associated with the dehydration of C-A-S-H (Figure 10b).

**Figure 11:**
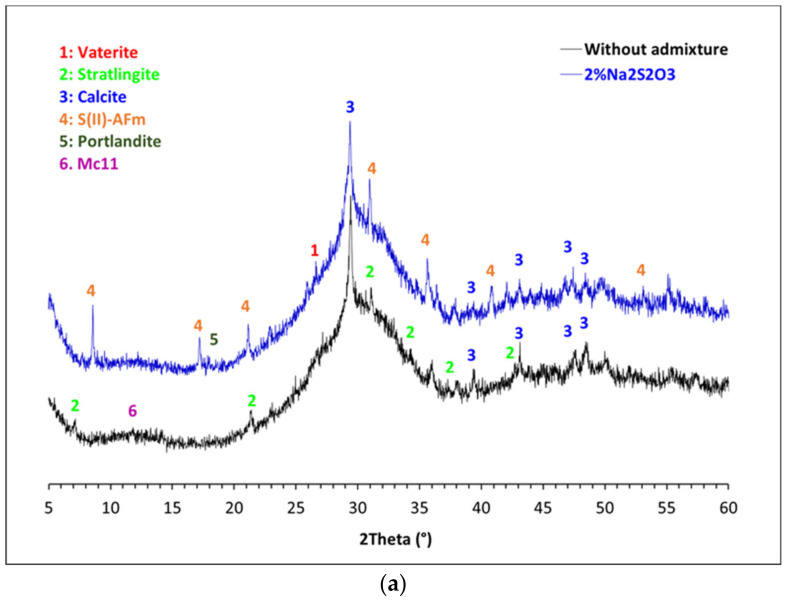

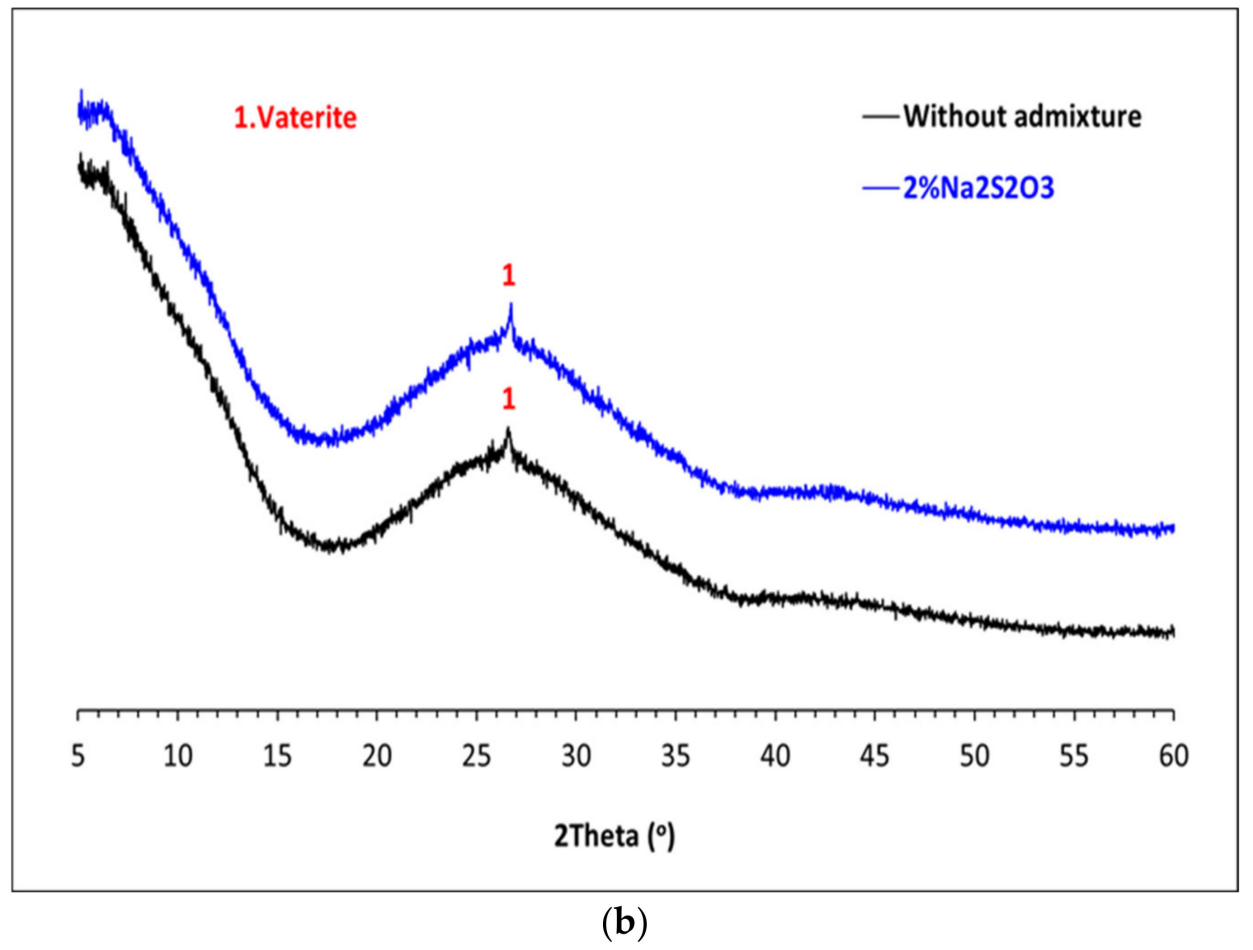
XRD difractograms of (**a**) 1 M NaOH- G1 and (**b**) 1 M NaOH- G2 pastes with and without 2%wt Na_2_S_2_O_3_ after 7 days of reaction.

**Figure 12:**
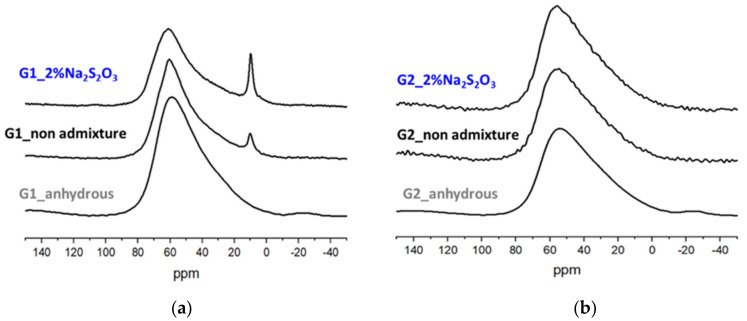
^27^Al NMR spectra of (**a**) G1 and (**b**) G2: anhydrous glasses and hydrated in absence or presence of 2% bwg Na_2_S_2_O_3_ after 7 days of hydration.

The addition of 2% Na_2_S_2_O_3_ induced the greatest change in the reactivity and mineralogy of 1 M NaOH- G1 and G2 pastes as observed from FTIR and TGA analyses. For this reason, these pastes were further investigated by XRD and ^27^Al MAS NMR techniques, and compared to plain samples. In non-admixed G1 pastes, the diffraction lines at 7° and 11.7° 2-theta (Figure 11a) after 7 days confirmed the formation of strätlingite [40] and monocarboaluminate [36], respectively, as previously observed by TGA (Figure 10a). In contrast, these phases were not formed in G1 pastes containing 2% Na_2_S_2_O_3_ (Figure 11a) while the presence of the diffraction lines at 8.5, 17.2 and 25.9° confirmed the formation of the S(II)-AFm phase [36]. In the ^27^Al MAS NMR spectra of G1 pastes (Figure 12a) with and without 2% Na_2_S_2_O_3_ two resonances at 61 ppm (4-fold Al) and 10 ppm (6-fold Al) were identified. The 4-fold Al corresponds to the Al incorporated in C-(N)-A-S-H and the initial glass. The 6-fold Al at 10 ppm is assigned to the AFm phases, strätlingite [41] and monocarboaluminate for the plain paste and S(II)-AFm for the admixed sample, in agreement with the XRD and TGA data. The higher intensity of the 6-fold Al peak for pastes containing Na_2_S_2_O_3_ indicates the higher amount of AFm phase formed in the admixed paste with respect to non-admixed sample, which agrees with the higher total chemically bound water measured for the former by TGA. The presence of calcite and vaterite in both G1 pastes indicated their partial carbonation.

In 1 M NaOH G2 pastes, with and without Na_2_S_2_O_3_, no crystalline reaction products (portlandite or AFm phases) are clearly detected by XRD (Figure 11b). The absence of 6-fold Al in the ^27^Al MAS NMR spectra (Figure 12b) confirms the absence of AFm phase in G2 pastes.

## 4. Discussion

In the first part of this paper, the influence of the chemical composition and structure of glasses and the solution composition (pH and admixtures) on their dissolution have been investigated. Higher dissolution rates have been calculated for the slag-type glasses (G1), with a lower polymerization (NBO/T = 1.58) with respect to Ca-rich fly ash glass (G2) (NBO/T = 0.33), in agreement with previous studies [25,42]. However, at the end of the dissolution tests, the degree of reaction of the slag-type glasses is slightly lower with respect to Ca-rich fly ash glasses despite the lower polymerization degree of the former. This could be explained for the different specific surface areas of both glasses (0.096 m^2^/g and 0.598 m^2^/g, for G1 and G-2, respectively, due to the less effective sieving of G-2 particles), a parameter that is not considered in the calculation of the degree of reaction (see Equation (4)).

In a pioneering study, Snellings [28] systematically showed that the dissolution of synthetic glasses with compositions similar to SCMs was controlled by the chemical composition of the solution. The presence of aluminates and calcium in solution inhibited the dissolution of pozzolanic glasses while Ca in solution only decreased the dissolution of slag-type glasses. In the current study, we moved one step forward and investigated the role of accelerating admixtures (0.02% TIPA, 0.02% DEIPA, 0.3% NaSCN and 0.3% and 2% Na_2_S_2_O_3_) on the glass reactivity. To our knowledge, this is the first time that this was done by investigating the dissolution of synthetic aluminosilicate glasses at far from equilibrium conditions (l/s = 1000) and on the reactivity of concentrated suspensions (l/s = 0.4). The obtained results inferred that the influence of these admixtures on the glass dissolution depends on the pH of the solution and the composition of the glass. In particular, in 0.1 M NaOH solutions (pH = 13.0), all the studied admixtures inhibited the dissolution of slag-type glasses probably due to their adsorption on glass reactive areas while TIPA, NaSCN and Na_2_S_2_O_3_ increased the rate of dissolution of the Ca-rich fly ash-type glass (see Table 3). The addition of 2% Na_2_S_2_O_3_, at this pH = 13.0, led to the highest increase of the degree of reaction of this glass from 4.2 to 6.2% after 48 h (an increase of around 50%). In contrast, in 1 M NaOH solutions (pH = 13.8), only the alkali admixtures (NaSCN and Na_2_S_2_O_3_) enhanced the degree of reaction of both types of glasses.

Different studies in the literature concluded that alkanolamines such as DEIPA, TIPA and TEA [16,17] increased the reactivity of aluminosilicates-SCMs in blended cements. However, the dissolution tests done in the current research concluded that DEIPA and TIPA did not have a significant impact on the dissolution and reactivity of the synthetic glasses. And even if the addition of TIPA to fly ash-type glass pastes led to an increase of the heat release from 15 to 26 J/g after 35 days of reaction (Figure 7), this was not linked to a higher amount of chemically bound water (Figure 8) and consequently a higher precipitation of reaction products. The disagreement of the effect of DEIPA and TIPA on synthetic glasses with respect to previous studies using SCM-blended cements could infer that the presence of minor elements in the real SCMs, such as Fe, or synergetic reactions between SCMs and OPC in blends could play a relevant role in the effect of these alkanolamines on the SCMs reactivity. Further studies on glasses with more complex compositions, including Fe, are required to fully understand the working mechanisms of TIPA and DEIPA on the reactivity of SCMs.

The kinetics studies done in pastes of glasses mixed with 1 M NaOH confirmed the higher impact of the accelerating admixtures on their reactivity. In particular, Na_2_S_2_O_3_ was the most effective admixture in slag-like systems leading to the highest increase of the total heat release (Figure 6) and chemically bound water (Figure 8a). TGA and XRD analysis (Figure 10a and Figure 11a) concluded that the addition of Na_2_S_2_O_3_ promoted the precipitation of the S(II)-AFm phase while monocarboaluminate and strätlingite were formed in free-admixtures pastes and in those containing DEIPA, TIPA and NaSCN. Furthermore, the amount of AFm formed in samples containing Na_2_S_2_O_3_ was significantly greater, with respect to non-admixed pastes, as inferred by the higher intensity of the 6-fold signal in the ^27^Al MAS NMR spectra. The consumption of a higher amount of aluminum from the pore solution to form this S(II)-AFm could enhance the further dissolution of the slag-type glass by two different mechanisms: (i) the lower passivation induced by the aluminates [33,43,44] and/or (ii) the increase of the undersaturation of the pore solution with respect to aluminum. Pustovgar et al. [33] proved that the adsorption of aluminates on the reactive sites of C_3_S hindered its dissolution, however, at pH higher than 13.0 calcium aluminate complexes formed in solution and did not adsorb onto the surface of the silicate. This would indicate that in slag-type glass pastes mixed with 1 M NaOH, with a pH = 13.8, adsorption of aluminates on the glass surface is not favourable and the increase of the aluminum undersaturation of the pore solution due to the precipitation of S(II)-AFm would explain the increase of the slag-like glass reactivity in presence of Na_2_S_2_O_3_. This AFm phase is not formed, at least initially (first 7 days), in Ca-rich fly ash glasses as shown by ^27^Al MAS NMR (Figure 12b) that could explain the lack of impact of Na_2_S_2_O_3_ on the hydration kinetics over the first 16 days (Figure 7). However, after this time, Na_2_S_2_O_3_ enhanced the dissolution Ca-rich fly ash glasses, although the reason of this delayed acceleration needs further investigation.

## 5. Conclusions and Outlook

In this paper, the impact of a series of accelerating admixtures commonly used in Portland cement (Na_2_S_2_O_3_, NaSCN, TIPA and DEIPA) on the dissolution of model synthetic glasses at high liquid to solid (l/s = 1000) and the reactivity of the corresponding pastes (l/s = 0.4) was investigated.

The effect of the accelerators on the glass dissolution depended on the pH of the NaOH solution and the glass structure. In 0.1 M NaOH (pH = 13.0), the accelerators inhibited the dissolution of slag-like synthetic glasses may be due to their adsorption on the glass surface, while they increased the dissolution of Ca-rich fly ash-like glasses. The alkali admixtures, NaSCN and Na_2_S_2_O_3_, led to the highest increase of the dissolution of both aluminosilicate glasses in 1 M NaOH (pH = 13.8).

Na_2_S_2_O_3_ has been identified as the most effective accelerator in slag-type glass pastes mixed with a 1 M NaOH, in which the addition of 2% Na_2_S_2_O_3_ enhanced the total heat release up to 55% with respect to non-admixed paste. In admixed and non-admixed slag-type glass pastes, C-(N)-A-S-H was formed, but while S(II)-AFm precipitated in samples containing Na_2_S_2_O_3_, monocarboaluminate and strätlingite were detected in non-admixed pastes. The higher amount of the AFm formed in presence of Na_2_S_2_O_3_ would increase the aluminum undersaturation in the pore solution that caused the greater dissolution of the glass.

In Ca-rich fly ash type pastes, both alkali accelerators led to the highest increase of heat release, but while the heat progressively increased for pastes containing 0.3% NaSCN, the addition of 2% Na_2_S_2_O_3_ only increased the total heat release after 16 days of reaction.

The studies described above have enabled us to identify Na_2_S_2_O_3_ as the most effective admixture to enhance the reactivity of synthetic aluminosilicate glasses at pH of around 13.8. The impact of this admixture on the reactivity, phase assemblage and mechanical behaviour of real blended cements should be now further explored.

Furthermore, the effect of these admixtures on glasses with a more complex composition, including Fe as a minor component, should be investigated. This would enable us to get a further understanding of possible Fe-TIPA complex formation, as concluded in the literature in the case of Portland cement [8,17], and its impact on glass reactivity.

## Figures and Tables

**Table 1:** Chemical composition (% mol) of the synthetic aluminosilicate glasses.

Glass Notation	CaO	SiO_2_	Al_2_O_3_	NBO/T
**G1**	47.29	41.62	11.09	1.58
**G2**	24.86	49.89	25.25	0.33

**Table 2:** Particle size and specific surface area of the synthetic phases.

Glass Type	SSA (m2/g)	Particle Size (µm)		
		Dv10	Dv50	Dv90
**G1**	1.08	1.77	13.28	42.03
**G2**	1.47	1.07	5.55	22.74

**Table 3:** Dissolution rate and degree of reaction of the synthetic glasses in 0.1 M NaOH and 1 M NaOH solutions with and without the accelerating admixtures.

Glass	NaOH Concentration	Parameter	Without Admixture	0.02% DEIPA	0.02% TIPA	0.3% NaSCN	0.3% Na_2_S_2_O_3_	2% Na_2_S_2_O_3_
**G1**	**0.1 M**	Log r_+Si_ (mol/m^2^/s) over first 6 h	−10.64	−10.64	−10.54	−10.68	−10.51	−10.73
		Glass reacted after 48 h (%)	5.26	5.02	4.23	4.32	4.13	4.14
	**1 M**	Log r_+Si_ (mol/m^2^/s) over first 6 h	−10.56	−10.76	−10.71	−10.65	−10.73	−10.57
		Glass reacted after 7 days (%)	7.43	5.55	7.35	7.97	5.93	7.73
**G2**	**0.1 M**	Log r_+Si_ (mol/m^2^/s) over first 6 h	−11.50	−11.47	−11.37	−11.43	−11.30	−11.26
		Glass reacted after 48 h (%)	4.23	4.66	5.78	5.45	4.53	6.22
	**1 M**	Log r_+Si_ (mol/m^2^/s) over first 6 h	−11.35	−11.19	−11.36	−11.28	−11.37	−11.33
		Glass reacted after 7 days (%)	8.46	7.44	7.98	9.89	8.65	9.88

## Data Availability

The data presented in this study and supplementary information are available on request from the corresponding author.

## Supplementary Materials

The following supporting information can be downloaded at: https://www.mdpi.com/article/10.3390/ma15030818/s1, Figure S1: X-ray powder diffraction (XRD) of (a) G-1 and (b) G-2 with and without the quenching method; Figure S2: (a) G-1 in 0.1 M NaOH; (b) G-1 in 1 M NaOH; (c) G-2 in 0.1 M NaOH and (d) G-2 in 1 M NaOH; Table S1: Saturation indexes of CNASH and Portlandite for after the dissolution test of G1 and G2 in far from equilibrium solutions in presence of the different chemical admixtures.
