# Supplementary material for: Influence of Accelerating Admixtures on the Reactivity of Synthetic Aluminosilicate Glasses
Source: Materials (Basel). 2022 Jan 21;15(3):818. doi: 10.3390/ma15030818 (PMC8836432; doi:10.3390/ma15030818)
Supplement: Supplementary file 1 [file materials-15-00818-s001.zip › materials-1536815-supplementary.pdf]

SUPPLEMENTARY MATERIAL

Influence of accelerating admixtures on the reactivity of synthetic aluminosilicate glasses

L. Gonzalez-Panicello, I. García-Lodeiro, F. Puertas, M. Palacios

Eduardo Torroja Institute for Construction Science (IETcc-CSIC), Spain

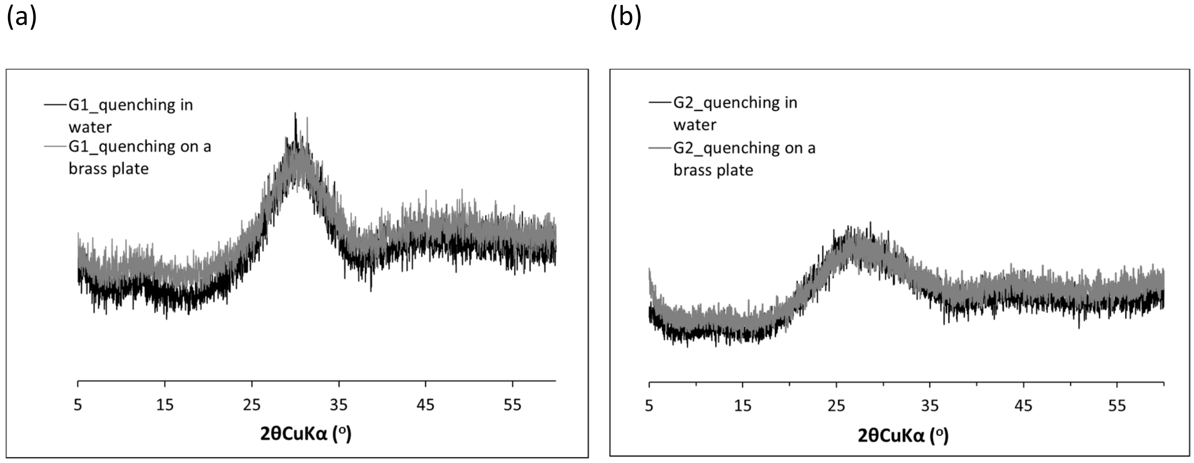

Figure. S1. X-ray powder diffraction (XRD) of (**a**) G-1 and (**b**) G-2 with and without the quenching method.

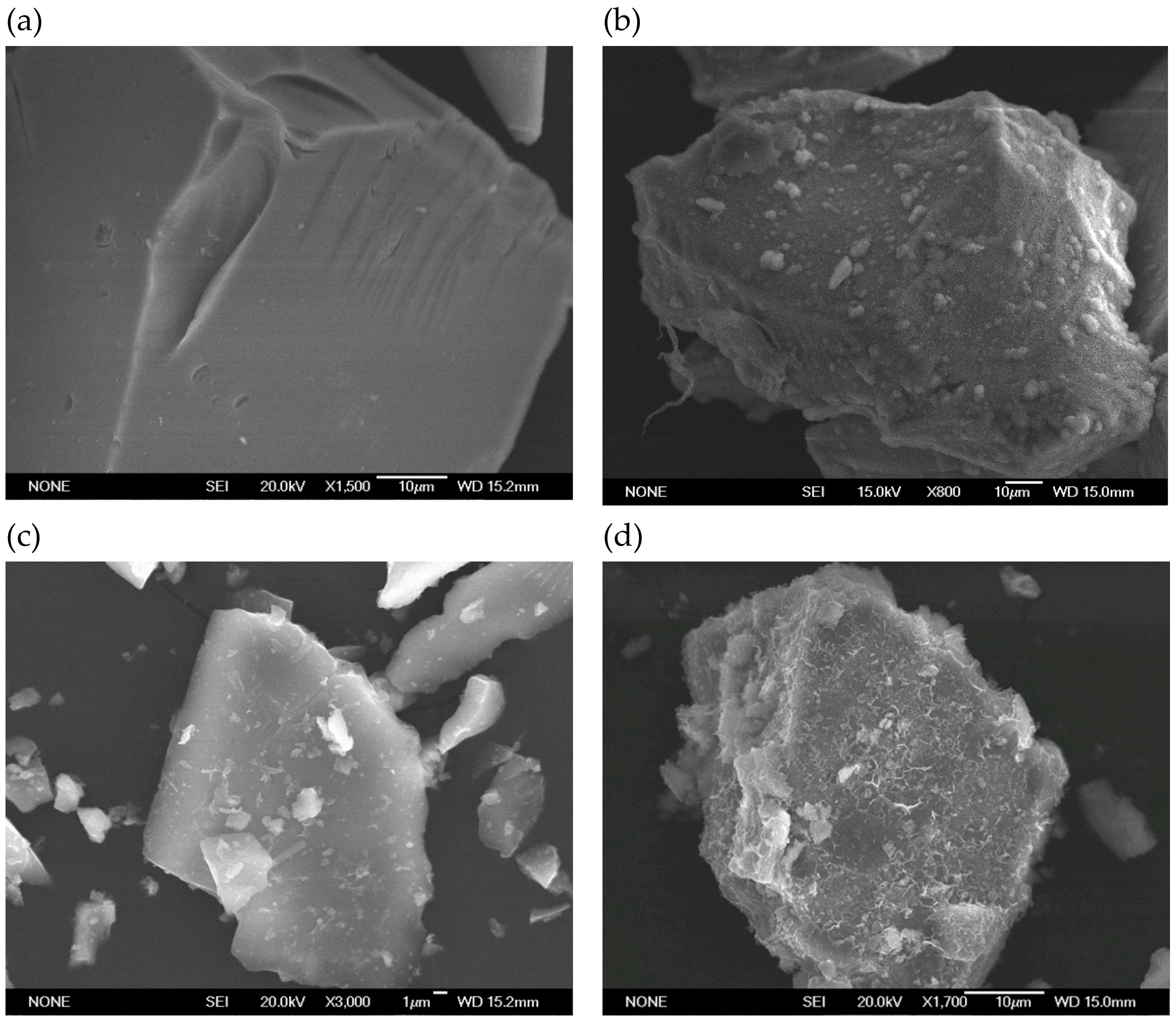

Figure S2. SEM images of (**a**) G-1 in 0.1M NaOH; (**b**) G-1 in 1M NaOH; (**c**) G-2 in 0.1M NaOH and (**d**) G-2 in 1M NaOH.

Table S1. Calculated saturation indexes of CNASH and Portlandite for after the dissolution test of G1 and G2 in far from equilibrium solutions in presence of the different chemical admixtures.

		**Solid**	**No admixture**	**0.02% DEIPA**	**0.02% TIPA**	**0.3% NaSCN**	**0.3% $Na_2S_2O_3$**	**2% $Na_2S_2O_3$**
**G-1**	**NaOH 0.1M**	CNASH	1.2929	1.2646	1.1580	1.1911	1.1984	1.0465
	**NaOH 1M**	CNASH	2.0398	1.1701	1.5555	1.7354	1.2281	3.8660
		Portlandite	0.1947	-	0.0829	0.1063	0.0007	0.1557
**G-2**	**NaOH 0.1M**	CNASH	1.0327	1.0377	1.0900	0.9564	1.0175	1.0196
	**NaOH 1M**	CNASH	1.0198	1.0482	1.3702	1.2736	1.0345	1.0668
